# Toward Understanding the Essence of Post-Translational Modifications for the *Mycobacterium tuberculosis* Immunoproteome

**DOI:** 10.3389/fimmu.2014.00361

**Published:** 2014-08-11

**Authors:** Cécile A. C. M. van Els, Véronique Corbière, Kaat Smits, Jacqueline A. M. van Gaans-van den Brink, Martien C. M. Poelen, Francoise Mascart, Hugo D. Meiring, Camille Locht

**Affiliations:** ^1^Centre for Infectious Disease Control, National Institute for Public Health and the Environment, Bilthoven, Netherlands; ^2^Laboratory for Vaccinology and Mucosal Immunity, Université Libre de Bruxelles (U.L.B.), Brussels, Belgium; ^3^Immunobiology Clinic, Hôpital Erasme, Université Libre de Bruxelles (U.L.B.), Brussels, Belgium; ^4^Institute for Translational Vaccinology, Bilthoven, Netherlands; ^5^Institut Pasteur de Lille, Center for Infection and Immunity of Lille, Lille, France; ^6^INSERM U1019, Lille, France; ^7^CNRS UMR8204, Lille, France; ^8^Université Lille Nord de France, Lille, France

**Keywords:** post-translational modification, *Mycobacterium tuberculosis*, CD4+ T cell epitope, proteomics, immunoproteome, T cell epitope repertoire, MHC ligands

## Abstract

CD4^+^ T cells are prominent effector cells in controlling *Mycobacterium tuberculosis* (Mtb) infection but may also contribute to immunopathology. Studies probing the CD4^+^ T cell response from individuals latently infected with Mtb or patients with active tuberculosis using either small or proteome-wide antigen screens so far revealed a multi-antigenic, yet mostly invariable repertoire of immunogenic Mtb proteins. Recent developments in mass spectrometry-based proteomics have highlighted the occurrence of numerous types of post-translational modifications (PTMs) in proteomes of prokaryotes, including Mtb. The well-known PTMs in Mtb are glycosylation, lipidation, or phosphorylation, known regulators of protein function or compartmentalization. Other PTMs include methylation, acetylation, and pupylation, involved in protein stability. While all PTMs add variability to the Mtb proteome, relatively little is understood about their role in the anti-Mtb immune responses. Here, we review Mtb protein PTMs and methods to assess their role in protective immunity against Mtb.

## Introduction

In the last few decades, the hallmarks of cell-mediated protection against *Mycobacterium tuberculosis* (Mtb), the causative agent of tuberculosis (TB), have been a subject of intense investigation. The production of the T helper cell type 1 cytokine IFNγ is considered key in Mtb immunity, since it is a central factor in activating macrophages to disarm intracellular mycobacteria (1, 2). A wide landscape of Mtb antigens targeted by human T cells is being uncovered, including proteins (3–6), lipoglycans (7–9), and lipoproteins (10–12) that are processed and exposed by antigen-presenting cells in the context of various presentation platforms. These can be either polymorphic classical MHC class I (HLA-A, -B, and -C) or MHC class II (HLA-DR, -DQ, and -DP) molecules (3–6, 10, 12), oligomorphic MHC class Ib molecules (HLA-E) (13–16) or CD1 isoforms (7–9, 11, 17–19). Relevant to the development of immunodiagnostic tests and vaccine candidates, strong human IFNγ responses consistently pointed at a range of immunodominant protein antigens, including members of the so-called PE/PPE and ESX protein families (5, 20–25). Whether these responses are for the greater part beneficial to the host by providing protection against Mtb or might actually help the pathogen to spread after damaging lung tissue is, for most of them, currently unanswered. Hyperconservation of human Mtb T cell peptide epitopes has been described, perhaps arguing for a beneficial effect of recognition by the host for the pathogen (26, 27), yet epitope sequence variability has also been reported (3, 28, 29).

Several genome-wide screens and bioinformatics-guided approaches further added to the identification of novel protein antigens and immunodominant epitopes for a number of antigen presentation platforms (5, 13, 24, 29–33). Altogether, the picture emerging from these studies is consistent with a multi-epitopic, multi-antigenic IFNγ response during Mtb infection. To investigate whether different protein classes have the same or diverse functional characteristics, Lindestam Arlehamn et al. combined genome-wide HLA class II binding predictions with high-throughput cellular screens of peptides to interrogate CD4+ T cell responses from latently infected individuals. A significant clustering was seen of the majority of targeted proteins, representing 42% of the total response to three broadly immunodominant antigenic islands, to only 0.55% of the total open reading frames (ORFs) (5). However, no quantitative, functional, or phenotypical distinction was observed between T cells elicited by the various protein classes involved, such as those assigned to be secreted or others belonging to secretion systems themselves, or to cell wall or cellular processes. Hence, because of equal functionality, no antigen class could be implied in a more protective (or non-protective) profile over others.

Even though greatly informative, preselecting epitope candidates from the full Mtb proteome of approximately 4,000 ORFs based on bioinformatics has limitations. Binding algorithms may not be 100% effective and certain protective Mtb epitopes with weaker binding properties could perhaps rank too low in the assignment to be selected.

Moreover, the assumption that the immunoproteome is merely a direct translation of the coding genome is an oversimplification. As an additional level of proteome complexity, primary protein structures can be modified after translation. Multiple post-translational modifications (PTMs) occur in higher and lower organisms, involving proteolytic events or transfer of modifying groups to one or more amino acids of the proteins. These PTMs may influence the protein’s active state, compartmentalization, turnover, and/or interactions with other proteins. The rich nature of PTMs of prokaryotic proteomes has started to become unraveled only recently (34), essentially through advances in mass spectrometry (MS) (35). However, their presence in the Mtb proteome and their role in virulence and immunity have not received sufficient attention yet. Here, we review PTMs currently known to occur in the Mtb proteome and discuss whether they modify the Mtb immunoproteome indirectly, by engaging eukaryotic innate receptor signaling or antigen-processing pathways, or directly by persisting as structural moieties in the immunogenic epitopes. In addition, we highlight technologies enabling the unbiased detection and identification of the Mtb T cell epitope repertoire, modified or unmodified.

## Post-Translational Modifications of Mtb Proteins

Current advances in MS-based proteomics have revealed that, like in eukaryotes, PTMs can create an enormous diversity and complexity of gene products in prokaryotes, as was reviewed recently elsewhere (34). PTMs are covalent-processing events chemically changing protein structure, often catalyzed by substrate-specific enzymes. Hundreds of types of PTMs are known, some of which can occur in parallel to create even more heterogeneity in the protein arsenal (36, 37). There are several technical obstacles still to overcome in PTM analysis. In proteome measurements, each protein can be identified based on combined mass and fragmentation patterns from various cleaved peptides. In PTM measurements, each modification site is only represented by a single peptide species. Modified peptides can be of low abundance and furthermore may have chemical properties requiring optimization of liquid chromatography (LC) separation techniques or fragmentation modules, used in MS identification. As a solution, robust MS-based proteomic workflows have been designed, including affinity-based enrichment strategies that can assist in the identification of, e.g., the phosphoproteome, the glycoproteome, or the acetylated proteome (35).

Over the last two decades, multiple proteomic studies were performed on Mtb. In one recent study, using dedicated subcellular fractionation combined with affinity enrichment and liquid chromatography mass spectrometry (LC-MS) based proteomics, Bell et al. were able to *bona fide* identify 1,051 protein groups present in the Mtb H37Rv proteome, including lipoproteins, glycoproteins, and glycolipoproteins (38). While data are accumulating, our insight into Mtb PTMs is still far from complete (see Table 1 for summary and structure examples of PTMs discussed).

**Table 1:** **Post-translational modifications in the Mtb proteome**.

PTM	Structure (example)	Function and notes	Mtb proteins exhibiting this PTM	Reference
targeted aa^a^				
ΔM^b^				
**O-glycosylation**		Pathogenesis	Apa/Rv1860; Mpt83/Rv2873; 19 kDa LpqH/Rv3763; 38 kDa PstS1/Rv0934; SodC/Rv0432; WGA enriched candidate glycoproteins	(38–45)
Thr, Ser	
		Immune decoy	
e.g. +162 (mannose)	
	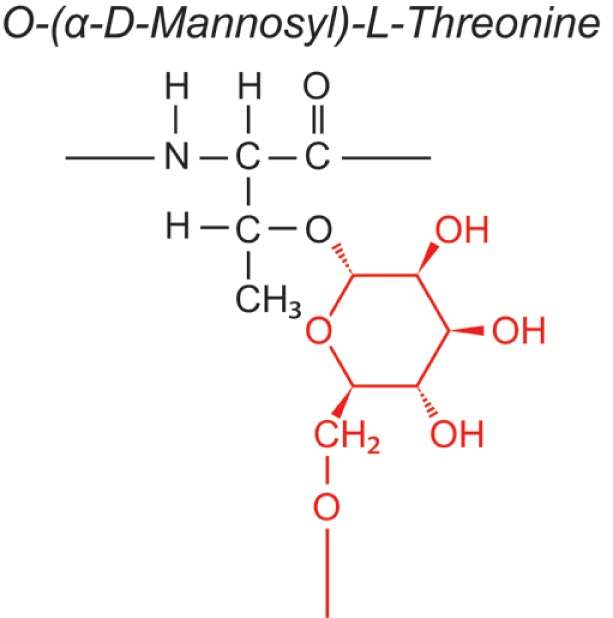			
**Phosphorylation**		Regulation	301 proteins	(46, 47)
Ser, Thr, Tyr	
+80	
	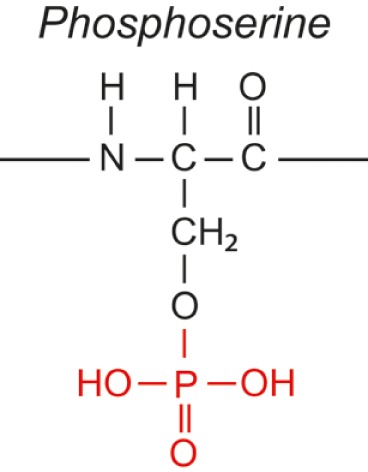			
**Methylation**		Protease resistance	HBHA/Rv0475; LBP/Rv2986c	(48)
Lys, Arg, Gln, Glu				
+28	
	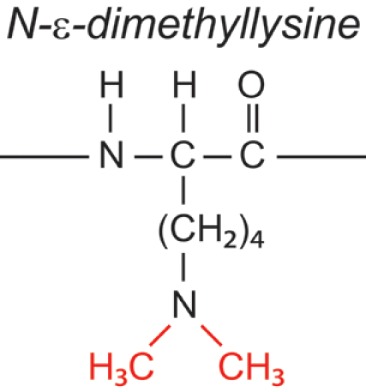			
**Acetylation**		Stability	Esat-6 (N-terminal threonine)	(49)
Ser, Thr, Lys		Compartmentalization	
(protein N-term)
+42	
	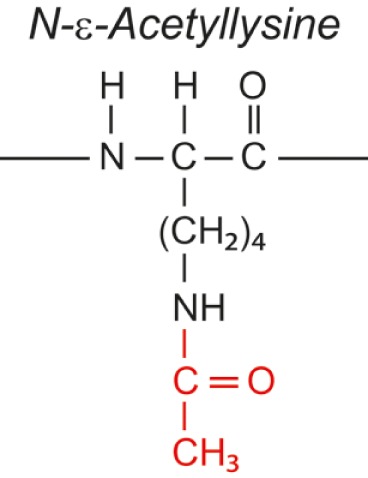			
**Lipidation**		Compartmentalization	99 Putative lipoproteins; 42 lipoproteins	(38, 42, 44, 50–55)
Cys, Ser, Thr		Anchoring in membrane	
+830	
	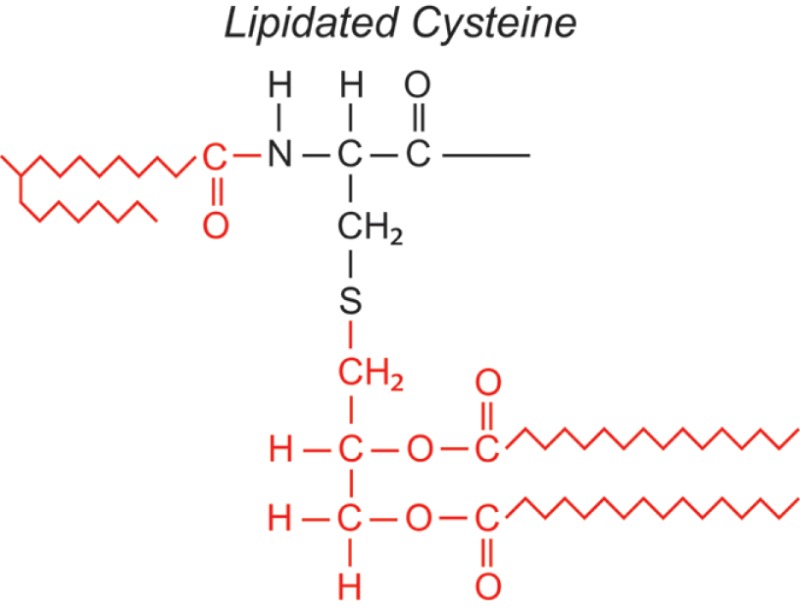			
**Deamidation**		Regulator of protein-ligand interaction	Pup/Rv2111c	(56)
Asn, Gln	
+1	
	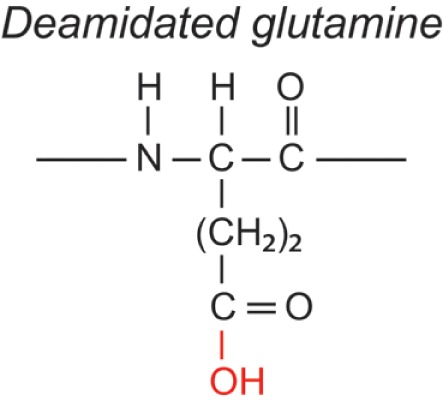			
**N-formylation^c^**		Start bacterial protein synthesis (fMet)	Rv0476, Rv0277C, Rv0749, Rv1686C	(57, 58)
Met (startcodon)	
+28	
	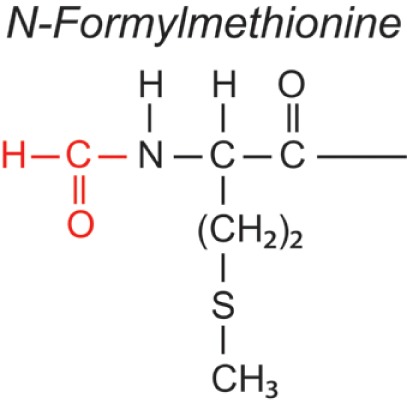			
**Pupylation**		Degradation signal (reversible)	1,305 proteins	(56, 59–64)
Lys	
+6,954	
	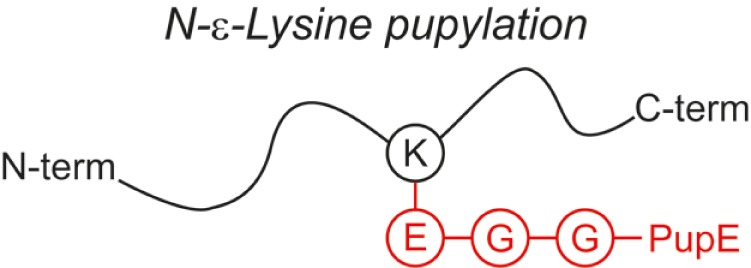			

*^a^aa amino acid*.

*^b^Mass increment of modified aa (Da)*.

*^c^Formally not a PTM but a modified aa*.

### Glycosylation

Prokaryotes possess conserved N- and O-linked glycosylation pathways, capable of enzyme-catalyzed covalently coupling glycans (oligosaccharides) to proteins (65–67). N-linked glycosylation, in which oligosaccharide precursors are first assembled on a cytoplasmic carrier molecule before being transferred *en bloc* to the amide nitrogen of an Asn in the acceptor protein, has not been observed in Gram-positive bacteria or in pathogenic mycobacterial species. O-glycosylation in bacteria can proceed *en bloc* or stepwise, but for Mtb it is thought to be the latter. A model was proposed in which the initial glycosyl molecule is transferred to the hydroxyl oxygen of the acceptor Thr or Ser residue, a process catalyzed by the protein *O*-mannosyltransferase (PMT) (Rv1002c) (39). Hereafter, further sugars are added one at a time, but the enzymes involved in this elongation are unknown. While the precise role of O-glycosylation of Mtb proteins is still elusive (68), this PTM appears essential for Mtb virulence, since Rv1002c deficient strains are highly attenuated in immunocompromised mice (69). Initially, glycoproteins of Mtb were reported to contain glycan moieties based on their ability to bind the lectin concanavalin A (ConA), e.g., 38 kDa (PstS1) protein (40). MS then enabled assessment of glycosylation patterns of Mtb proteins, first the alanine-proline-rich 45–47 kDa antigen Apa (41, 70), followed by others, e.g., the lipoproteins (19 kDa) LpqH (42, 43) and SodC (44). Using ConA affinity capture or other sugar-based partitioning methods, and dedicated proteomics, Bell et al. reported a wealth of candidate Mtb glycoproteins, associated with membrane fractions and with culture filtrates (38), whereas others, comparing several fragmentation strategies, identified novel glycosylation sites directly from culture filtrate proteins (45, 71). These localizations corroborate with data suggesting that O-glycosylation and Sec-translocation, a process shuttling proteins across the bacterial cell envelope, are linked (39). As the number of *bona fide* identified Mtb glycoproteins is increasing, a glycosylation site motif is emerging, frequently observed at the protein C-terminus (45). Some O-glycosylated Mtb proteins constitute B cell antigens for serodiagnostics, such as the 38 kDa protein (72). Furthermore, they might contribute to the virulence of Mtb by binding as adhesins to innate immune receptors, promoting invasion of the host cells. The 19-kDa glycolipoprotein was shown to bind to the macrophage mannose receptor (MR) of monocytic THP-1 cells, hereby promoting the uptake of bacteria (73). Apa, secreted, as well as cell wall associated, binds to human pulmonary Surfactant Protein A (SP-A), an important lung C-type lectin (74). These two glycoproteins were also reported to be involved in Mtb binding to DC-SIGN on dendritic cells, although this needs further investigation (75).

### Phosphorylation

Since Mtb can exist under various physiological states in the host, including dormancy and active replication, it makes use of a versatile mechanism to sense signals from the host and regulate cellular processes. Signal transduction through reversible protein phosphorylation participates in this function. The Mtb genome encodes multiple serine/threonine protein kinases, and Ser/Thr/Tyr protein phosphorylation occurs extensively. In addition, Mtb makes extensive use of two-component signal transduction systems, which rely on a phosphorylation cascade involving His kinases (46). Using TiO_2_-phosphopeptide enrichment, Prisic et al. assigned 301 phosphoproteins in Mtb grown under six different conditions and identified corresponding phosphorylation site motifs (47). These likely represent only a part of the Mtb phosphoproteome. However, little is known on the role of this PTM in the function or pathogenicity of these proteins, with exception of the His kinases in two-component systems (46).

### Lipidation

Lipidation of proteins is predicted for a small percentage (0.9–2.5%) of ORFs in mycobacterial genomes, and is required for their anchoring and sorting to the cell surface [reviewed in Ref. (50, 76)]. The first step in Mtb lipoprotein biogenesis occurs in the N-terminal leader of preprolipoproteins having a so-called lipobox motif, involving the attachment of diacylglycerol to the thiol group of a Cys, by Lgt (phosphatidylglycerol-pre-prolipoprotein diacylglyceryl transferase). Second, the signal peptide directly upstream of the modified Cys is cleaved off by LspA (prolipoprotein signal peptidase/signal peptidase II). Only recently, proof was found that slow-growing Gram-positive mycobacteria also share the third step in lipoprotein biosynthesis with Gram-negative bacteria, i.e., adding a third acyl residue to the free amino group of the modified Cys by Lnt (phospholipid-apolipoprotein *N*-acyltransferase) (51). Brulle et al. described the BCG_2070c as the major ORF in BCG to encode a functional Lnt using a mycobacteria-specific acyl substrate, tuberculostearic acid (52). Lipoprotein genesis is essential for Mtb. Deletion of lgt was not possible (77), while an lspA deletion mutant was viable but had an attenuated phenotype (78, 79). For Mtb, multiple (candidate) lipoproteins have been identified, and classified as components of transport systems, enzymes, or as molecules involved in cell adhesion or in signaling (38, 50), several of which were not only lipidated but also glycosylated (42, 44, 52). In line with the dogma that lipoproteins are pathogen associated molecular patterns (PAMPs) sensed by TLR2 (80), Sanchez et al. showed that the glycolipoprotein 38 kDa PstS1 triggers a TLR2 and caspase-dependent apoptotic pathway in human macrophages (53). Besides this mechanism, the 19-kDa glycolipoprotein LpqH was shown also to induce a caspase independent apoptotic mechanism, involving mitochondrial apoptosis-inducing factor (AIF), killing macrophages (54). Furthermore, TLR2-dependent inhibition of MHC class II function was observed for LpqH (81). The cumulative data on LpqH suggest that through its PTMs, this glycolipoprotein exploits multiple innate immune receptors and mechanisms to enter (73), incapacitate, and kill mononuclear phagocytes. Notably, Lopez et al. reported that the lipid moiety of LpqH was not required for the TLR2-dependent apoptosis of macrophages (82). As another innate feature, LpqH and the lipoprotein LprG were found to directly stimulate TLR2/TLR1 on memory CD4^+^ T cells (55), presumably via engaging TLR2 and TLR1 pockets by their thioether-linked diacylglycerol and amide-linked third acyl chain, respectively (83).

### Formylation

Formylation/de-formylation of proteins is a typical hallmark of bacterial proteomes. Protein synthesis in bacteria is initiated with a formylated methionine (fMet) residue, which is then enzymatically cleaved by peptide deformylase (PDF) and methionine aminopeptidase to generate mature proteins. The human immune system can benefit from this unique formylation pathway to distinguish self from non-self proteins. Although formylation is not strictly a PTM, but comes with the first “modified” building block of protein synthesis, the presence of the formyl group can be considered a variation of plain translation of the genetic code. What might be the life span of the formylated state of proteins is unknown so far. However, short formylated Mtb protein fragments have been identified that can be presented as epitopes via non-classical murine MHC class Ib molecules of infected macrophages and appear to be protective in a Mtb challenge model (57, 58). This suggests that *in vivo*-formylated proteins can enter antigen-processing pathways before the enzymatic removal of the N-terminal fMet residue has occurred. Recently, N-formylated peptides of ESAT-6 and glutamine synthetase were found to have immunotherapeutic potential in a Mtb mouse infection model. A role for formyl peptide-receptor recognition in activation of innate immune cells was implied (84), but presentation via non-classical MHC molecules may also play a role.

### Pupylation

Pupylation is a protein-to-protein modification, first identified in Mtb. It covalently attaches the C-terminal Glu of the 6.9-kDa “Protein Ubiquitin-like Protein” (Pup) to the ε-amine of Lys side chains of an interacting protein partner (59). Although the full purpose of the pupylation pathway in Actinobacteria remains to be elucidated, it is assumed that in Mtb, disposing of a proteasomal system, tagging proteins with Pup renders them susceptible for proteasomal degradation (60–62), similar to the well-known ubiquitin-initiated protein degradation pathway. The C-terminal Glu of Pup itself is generated by another PTM, i.e., deamidation of the C-terminal Gln (56). From various large-scale proteomic studies, a database of the mycobacterial “pupylome,” containing > 150 verified pupylated proteins and >1,000 candidate pupylated proteins, was annotated (63). Depupylation activity also occurs (64), hence the modification can be reversed.

### Acetylation and acetyl-like modifications

Transferring an acetyl, propionyl, maloyl, or succinyl group to the ε-amine of lysines (N^ε^-modification) or to the α-amines of protein N-termini (N^α^-modification) are widely occurring PTMs in prokaryotes (34). Mtb encodes multiple proteins annotated as putative acetyl transferases acting on protein substrates (85). A well-studied N^α^-acetylated Mtb protein is the virulence factor and immunodominant antigen, early secretory antigenic target 6 (Esat-6) (49). Acetylation presumably confers protein stability and compartmentalization, and occurs at Thr2, becoming the N-terminus after removal of the fMet residue at position 1.

### Methylation

**Figure 1:**
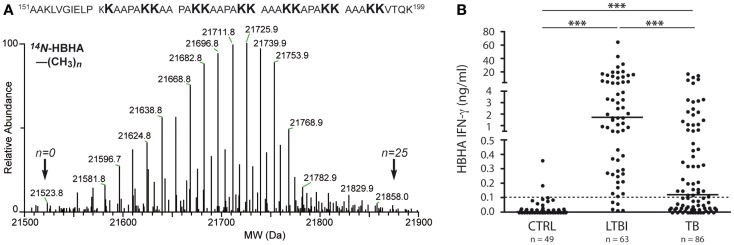
**Molecular and immunological hallmarks of naturally methylated HBHA**. **(A)** LC-MS analysis (lower part) and summary of methylation pattern (upper part) of HBHA from BCG. Indicated by arrows are the masses of molecular variants in the mass envelope, the lowest and highest of which correspond to HBHA containing 0 or 25 methyl groups, respectively. Methylations are borne by the lysine residues of the C-terminal part. Data indicate that at least 13 out of the 16 C-terminal lysines can be mono- or dimethylated. **(B)** *In vitro* IFNγ release to methylated HBHA stimulation according to Mtb infection status. Shown are IFNγ concentrations in nanogram/milliter as measured in Elisa after stimulation with methylated HBHA for 24 h of PBMC from three groups of subjects: non-infected controls (CTRL), subjects with latent Mtb infection (LTBI), and patients with active tuberculosis (TB). The dotted line represents the positivity cut-off for the assay. For each group, the median of results is marked as a horizontal line. Statistical significance of differences: ***p ≤ 0.0001. Data are with licensed permission from Ref. (23).

This PTM involves the addition of one or several methyl groups to either the ε-amine of lysines or to the side chain carboxyl of Glu. Although this PTM occurs in Mtb, genes encoding Mtb protein-methyltransferases have not been identified yet. Two Mtb adhesins, heparin-binding hemagglutinin (HBHA, Rv0475) and laminin-binding protein (LBP, Rv2986c) were shown to be methylated (48). HBHA is a 28-kDa multifunctional protein found on the surface and in culture filtrates of mycobacteria. Automated Edman degradation and mass spectrometric analysis indicate that at least 13 out of 16 Lys residues in the Lys-Ala-Pro rich C-terminal region of HBHA can be mono-or dimethylated, generating a spectral envelop of isoforms (Figure 1A) (48). HBHA mediates mycobacterial adherence to epithelial cells via the interactions of this C-terminus with sulfated glycoconjugates on the surface of epithelial cells and methylation was implied to play a role in resistance to proteases present in bronchoalveolar lavage fluids (86–88). Recently, Sohn et al. showed that HBHA from Mtb also targeted murine macrophages and induced apoptosis via a mechanism involving mitochondria (89). Interestingly, HBHA purified from *Mycobacterium avium* subsp. *paratuberculosis* contains an N-terminal acetylated alanine residue in addition to the methylated lysines (90), whereas there is no evidence for acetylation of the N-terminal residue of Mtb HBHA (88).

## Post-Translational Mtb Protein Modifications in Protective Immunity and Vaccine Candidates

The rich variety of PTMs to a large proportion of the Mtb proteome is likely to play a major role in the successful intracellular lifestyle of Mtb during chronic and sometimes lifelong infections. In the quest of novel vaccines, urgently needed to improve the limited protective capacity of BCG, it may be useful to understand the role of these PTMs in the host response to Mtb infection. Over thousands of years, a balance has been reached in which Mtb avoids excessive immunity allowing it to survive in the host, and in which a certain level of immunity allows the host not to succumb to the infection.

While the primary Mtb proteome shows features of hyperconservation, suggesting an evolutionary advantage to ensure stable epitope recognition by CD4+ T cells (26), PTMs superimpose a high level of complexity. This may complicate the identification of protective protein antigens based on *in silico* analyses and recombinant DNA technologies. Once protective protein antigens have been identified, the exact structural features need to be known for optimization and process development of the antigen. Furthermore, it will be important to know whether a particular PTM acts as an immune modulator, or/and whether it is part of the structural antigen moiety targeted by the adaptive immune system. Illustrative in this respect are three examples of Mtb protein antigens with PTMs, currently considered as vaccine candidates because of their immunodominance in humans and/or protective effect in animal models.

The 45–47 kDa secretory and cell-surface adhesin Apa is a major mycobacterial antigen with different O-mannosylation patterns in pathogenic versus non-pathogenic mycobacterial species that are critical for its T cell antigenicity *in vivo* and *in vitro* (70, 91). T cells from BCG-vaccinated PPD-responsive individuals recognize either both native mannosylated Apa (nApa) and recombinant non-mannosylated Apa (rApa), or nApa only. These latter T cells did, in contrast to the former, not recognize synthetic peptides corresponding to the Apa protein sequence. Together with the finding that recognition of nApa required active antigen processing, these data suggest that mannosylation does not induce alternate processing of nApa but rather that the carbohydrate moiety is an intrinsic part of the T cell epitope(s) (92). Protection by Apa was shown in guinea pig and mouse models in the context of various vaccine platforms (protein, DNA, and poxvirus boost) and routes (intanasal and subcutaneous), as a subunit or as a BCG-booster vaccine (70, 92–94). In a mouse model, adjuvanted nApa was found to induce higher frequencies of CD4+ T cells, producing more cytokines, compared to adjuvanted rApa. However, both antigens were equally protective against virulent Mtb infection when used as a subunit vaccine or as a BCG-booster vaccine (92). This indicates that O-mannosylation is not required for the protective effect in this model. However, understanding of the impact of the different immune responses evoked by nApa and rApa, as well as the nature of the putative naturally processed glycopeptide(s), need further investigation.

In contrast to Apa, the natural PTM of HBHA, methylation, is essential for providing high levels of protection against Mtb challenge in mice, in addition to its antigenicity in Mtb-infected human individuals (95, 96). However, immunization of mice with purified non-methylated HBHA induces antibodies and Th1 cytokines at levels similar to those induced by immunization with methylated HBHA. Also, the antibody isotype profiles are similar in both instances. Interestingly, however, only splenocytes isolated from mice immunized with methylated HBHA, and not with non-methylated HBHA, induce IFNγ secretion upon incubation with Mtb-pulsed macrophages. Methylated HBHA-specific T cell responses are likely to participate in protection against disease in humans, since T cells from patients with active TB secrete significantly lower amounts of IFNγ after stimulation with methylated HBHA than subjects with latent Mtb infection (Figure 1B) (23, 97, 98). HBHA is being considered as a BCG-booster vaccine (99), as responses to methylated HBHA were found to be primed in BCG-vaccinated infants (100). It is not yet known whether the PTM affects the presentation of non-modified protective T cell epitopes via modulation of antigen uptake or processing, or whether methylation is part of the protective T cell epitope(s) involved.

The N-terminal-Thr acetylated antigen ESAT-6 is known as an immunological hotspot in humans (6). During natural infection or after subunit vaccination in mice, vigorous Th1 type CD4^+^ T cell responses are directed to the N-terminal immunodominant epitope ESAT-6_1–15_, whereas other epitopes are masked (101). These can be revealed by redesigning ESAT-6 analogs in which the dominant epitope is removed, resulting in the engagement of protective CD4^+^ T cell responses that resist infection-driven terminal differentiation (102). To our knowledge, the role of the N-acetylation at Thr2 in generating the ESAT-6 peptide repertoire has not been interrogated, yet in view of ESAT-6’s current status as a vaccine candidate in clinical testing (99, 103), such assessment may be important.

In order to fully characterize these candidate vaccine antigens, it will be important to elucidate the exact roles of the added glyco-, methyl-, or *N*-acetyl moieties, respectively. Does their presence modulate effective antigen processing, perhaps by steering proteolysis and immunodominance through masking certain enzyme cleavage sites as was shown for O-linked glycans (104), or are they part of the protective immunoproteome itself ? Clearly more studies are needed, including epitope identification approaches to unravel, in these and other targeted vaccine candidates, the role of PTMs in the Mtb immunoproteome. Knowledge on the precise role of the PTM of Mtb vaccine candidates may be of great help to optimize vaccine candidates and potentially to simplify vaccine design and process development.

## Toward Unbiased Assessment of the Mtb Immunoproteome

Protein antigens, modified or not, are translated for T cell surveillance into immunogens in antigen-processing pathways of antigen-presenting cells. This translation consists of enzymatic cleavage and rescue of protein fragments onto the molecules of a relevant antigen-presenting platform, such as classical class I or II MHC molecules (105), non-classical MHC molecules, including class Ib MHC molecules (16), or CD1 isoforms (17). The identification of the exact nature of the naturally processed and presented Mtb immunoproteome would require dedicated technologies such as LC-MS, first pioneered MHC class I ligands by Hunt et al. more than two decades ago (106, 107). Typically, cell lines would be grown at large scale (>1 × 10^9^ cells) and, after detergent solubilization and immunoaffinity purification of MHC-ligand complexes, bound peptide epitopes would be eluted. The purified endogenous MHC class I ligands were characterized by dedicated LC-MS and MS/MS sequencing.

Nowadays, ever evolving LC-MS/MS systems have greatly added to our understanding of the endogenous peptide repertoire and binding motifs of many MHC class I and II molecules (108–111), as well as of class1b MHC molecules (112). For the classical MHC pathways, the notion has emerged that antigen-presenting cells express approximately 100,000 MHC class I and II molecules at their surface, presenting thousands of different endogenous peptides, at widely divergent abundances (113). LC-MS/MS sequencing can unambiguously identify the epitopes as they are eluted from their antigen-presenting molecules in a qualitative and quantitative manner, revealing both primary epitope sequences, as well as any modifications to them (114). LC-MS/MS analyses have shown that processing inside antigen-presenting cells can generate modified or unpredictable MHC epitopes, such as deamidated (115), citullinated (116), or cysteinylated (117) ligands, as well as ligands arising from protein splicing (118–120) or from alternative reading frames or read-throughs of protein-encoding genes (121–123).

Pathogen-encoded immunoproteomes, including PTMs, generated from the proteome inside infected or antigen endocytosing antigen-presenting cells, should be detectable through LC-MS/MS sequencing approaches as well, although pathogen-derived ligands will be needles in the haystack of eluted *self* epitopes. To facilitate the identification of these *non-se*lf pathogen-derived antigens, targeted LC-MS/MS approaches have been developed (124–127). Foreign epitopes that originate from proteins synthesized during infection inside antigen-presenting cells, such as viral MHC class I epitopes during infection, can be traced using algorithms detecting isotopic patterns in the mass chromatograms of MHC immunoproteomes from carefully mixed infected and non-infected cell cultures that were metabolically labeled during growth (128). Alternatively, epitopes that arise from exogenous proteins endocytosed by antigen-presenting cells during infection, such as bacterial MHC class II epitopes, can be traced back in the MHC-bound peptide repertoire after metabolic labeling of antigen during the prokaryotic cell growth (126, 129). However, if PTMs are suspected in the foreign MHC immunoproteome, chromatography, ion fragmentation strategy, and even affinity enrichment strategies will have to be considered accordingly. Until now, only a single study has reported the identification of several Mtb epitopes presented by MHC class I via LC-MS (130). More approaches are underway to extend our knowledge on the naturally processed and MHC-presented Mtb epitopes, including those derived from methylated HBHA, using dedicated LC-MS. These studies include large-scale human monocyte or dendritic cell cultures and either *in vitro* Mtb infection or targeted antigen pulsing. Inhibition of MHC class II presentation upon incubation with live Mtb, mycobacterial lysates, or purified antigens may frustrate these attempts (131, 132). Dedicated isolation and analytical discovery procedures should then help to identify the Mtb epitope “needles” in the *self* “haystack,” and increase our knowledge on the role of PTM in the Mtb immunoproteome.

## Concluding Remarks

Fast developments in LC-MS/MS-based proteomics have enabled the detection of many types of PTMs in proteomes of prokaryotes, including Mtb. Elucidating the role of PTMs in the immunoproteome of protective Mtb protein antigens is important for the molecular optimization of vaccine candidates, and will also greatly benefit from technical advancements in LC-MS/MS.

## Conflict of Interest Statement

The authors declare that the research was conducted in the absence of any commercial or financial relationships that could be construed as a potential conflict of interest.
